# Exploring the Oral Resistome: From Metagenomics to Precision Oral Health

**DOI:** 10.3390/microorganisms14092033

**Published:** 2026-09-12

**Authors:** Ludovic Nunes Correia, Adelina Correia, Lucinda J. Bessa

**Affiliations:** Egas Moniz Research for Interdisciplinary Research (CiiEM), Egas Moniz School of Health & Science, 2829-511 Caparica, Almada, Portugal

**Keywords:** antibiotic resistance genes, functional resistome, genetic resistome, multi-omics integration, oral microbiome, oral resistome, precision dentistry, shotgun metagenomics

## Abstract

Antimicrobial resistance (AMR) represents one of the foremost global public health threats, undermining the efficacy of antibiotic therapies across all clinical disciplines, including dentistry. The oral cavity, housing one of the most diverse microbial ecosystems in the human body, contains a largely underappreciated reservoir of antibiotic resistance genes (ARGs), collectively defined as the oral resistome. This review synthesises current evidence on the oral resistome across five thematic areas. First, we contextualise the global burden of AMR, highlighting its scale and implications for oral healthcare. Second, we define the oral resistome and characterise its composition, distribution across oral microhabitats, and the principal determinants that govern its structure and dynamics. Third, we critically appraise metagenomic approaches, from early culture-based and PCR-targeted methods to shotgun metagenomics and functional screening, that have expanded the resolution of resistome characterisation. Fourth, we examine multi-omics integration, including genomics, transcriptomics, and metabolomics, and its capacity to reveal the ecological and molecular drivers of resistance within the oral ecosystem. Finally, we explore how resistome profiling can inform precision oral health, enabling individualised antimicrobial stewardship, microbiome-based risk stratification, and patient-tailored preventive and therapeutic strategies in the era of personalised dentistry.

## 1. Introduction

The oral cavity harbours one of the most diverse and complex microbial communities in the human body, comprising bacteria, archaea, fungi, protozoa and viruses that collectively constitute the oral microbiome [1,2,3]. Among the bacterial taxa, *Streptococcus* is the predominant genus. Species belonging to the *Streptococcus mitis* group are among the earliest colonizers of the oral cavity in newborns and act as primary colonizers, creating conditions that promote the establishment and development of a complex oral microbial community [4,5,6]. The anatomical complexity of the mouth shapes its microbial diversity: distinct environmental conditions across teeth, gingival sulcus, tongue, cheek mucosa, and hard and soft palate create multiple micro-habitats that support structurally organized and distinct, biofilm-based microbial communities [2,6]. The oral microbiome is not merely a passive resident but plays an active role in maintaining local and systemic health, and its disruption has been implicated in a range of oral and extra-oral diseases [3,7,8].

A growing body of evidence indicates that this microbial community also functions as a substantial, largely unappreciated reservoir of antimicrobial resistance. Resistance in oral bacteria is frequently conferred by antibiotic resistance genes (ARGs), and the collection of these genes across the oral microbiota has come to be described as the oral resistome [9]. Genetic surveys using metagenomic approaches have revealed that ARGs, including previously undescribed variants, are widely distributed even among individuals with no recent history of antibiotic exposure, prompting a conceptual shift from tracking resistance in individual pathogens toward characterising resistance as a community-wide property of the oral ecosystem [10,11]. Notably, next-generation sequencing (NGS)-based metagenomic studies consistently detect substantially more ARGs and ARG-carrying taxa than PCR-based approaches, underscoring the value of untargeted sequencing for capturing the full extent of resistance gene diversity, including in bacterial taxa that remain difficult or impossible to culture [9,12].

At the same time, the broader field of precision medicine has begun to reshape dentistry. The integration of high-throughput “omics” technologies, including genomics, transcriptomics, metabolomics, and microbiome profiling, into clinical dental practice is enabling more individualised approaches to diagnosis, risk prediction, and treatment planning, an approach increasingly referred to as precision dentistry [13]. Saliva has emerged as a promising diagnostic biofluid for microbiome, proteome, and genomic profiling, facilitating individualized health assessment and monitoring [14]. Applications range from salivary biomarker-based diagnostics for periodontal disease and caries risk to microbiome-informed antimicrobial stewardship and pharmacogenomically guided drug regimens [13].

**Figure 1:**
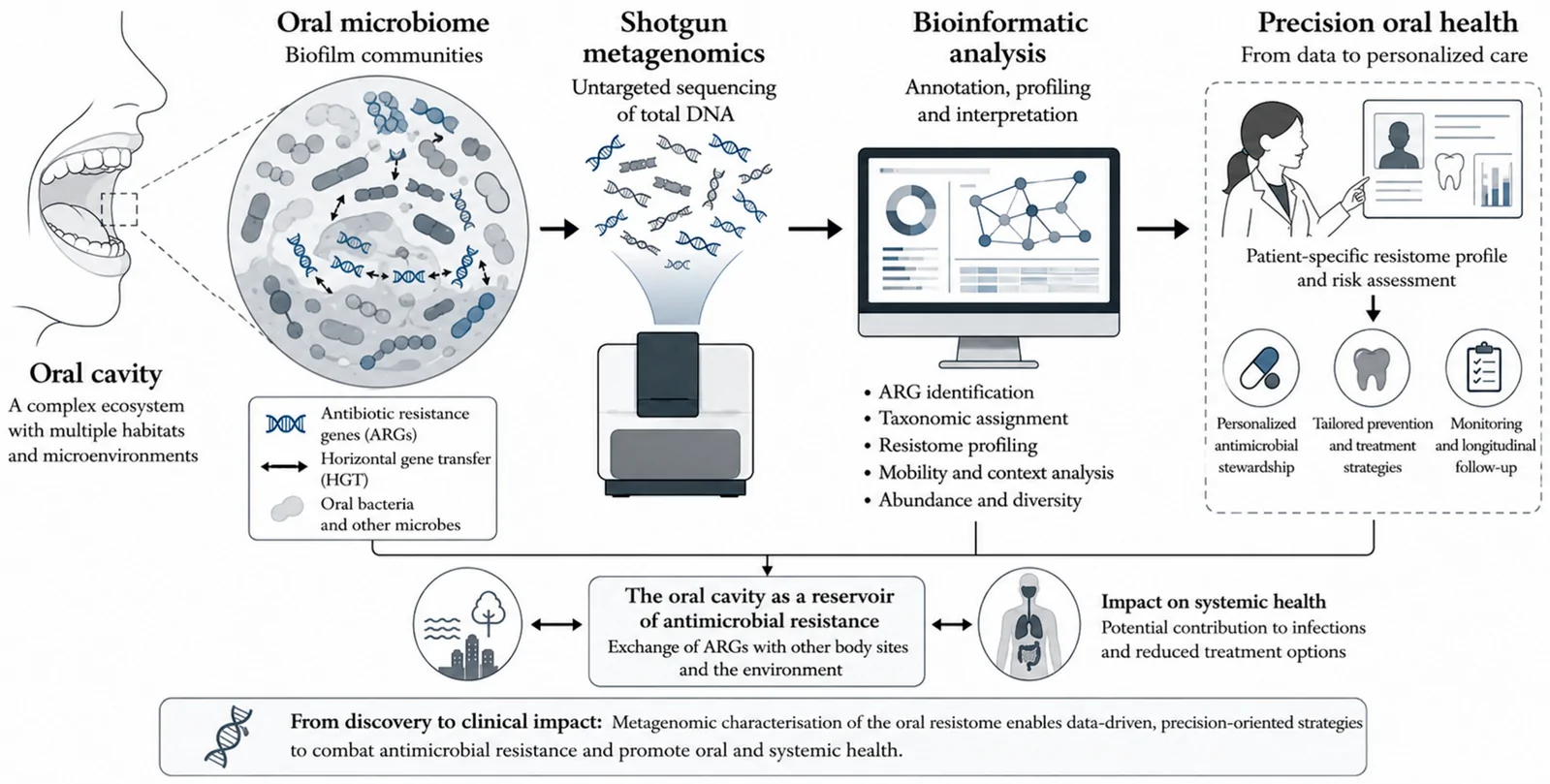
Conceptual overview of the oral resistome and its role in precision oral health. The oral cavity harbours a diverse biofilm-associated microbiome that serves as an important reservoir of antimicrobial resistance genes (ARGs). Horizontal gene transfer facilitates the dissemination of ARGs among oral microorganisms, contributing to the establishment of the oral resistome. Shotgun metagenomic sequencing, combined with bioinformatic analysis, enables comprehensive identification and characterization of resistance genes, their microbial hosts, and their mobility potential. Integration of resistome profiling into precision oral health supports patient-specific risk assessment, antimicrobial stewardship, personalized preventive and therapeutic strategies, and longitudinal monitoring, while highlighting the role of the oral cavity as a potential source of antimicrobial resistance dissemination with implications for both oral and systemic health.

Within this context, characterising the oral resistome through metagenomic sequencing represents a natural extension of precision dentistry (Figure 1): understanding the distribution, mobility, and drivers of resistance genes within an individual’s oral microbiome could inform more judicious, patient-specific use of antimicrobials and support early detection of resistance risk before it manifests clinically [15].

This review therefore aims to synthesise current knowledge on the oral resistome, tracing its characterisation from early PCR-based detection methods to contemporary shotgun metagenomic and functional metagenomic approaches, and to examine how this growing body of evidence might be translated into precision-oriented strategies for oral health management.

## 2. Literature Review Methodology

The studies considered in this review were identified through searches of several bibliographic databases, with PubMed and Scopus serving as the primary sources. The literature search employed a range of keywords and combinations thereof, including oral microbiome, antibiotic resistance, oral resistome, antibiotic resistance genes, functional resistome, genetic resistome, multi-omics, precision dentistry, and metagenomic approaches. No restrictions were imposed regarding article type, and no predefined inclusion criteria were established. Instead, the relevance of each retrieved publication was assessed by the authors, with the aim of incorporating a broad, balanced, and up-to-date body of evidence. The majority of the studies included in the review were published from 2015 onwards.

## 3. The Oral Microbiome as a Reservoir and Disseminator of Antimicrobial Resistance

### 3.1. The Oral Microbiome as a Reservoir of Antimicrobial Resistance

AMR has emerged as one of the most pressing threats to global public health in the 21st century. A landmark systematic analysis estimated that, in 2019 alone, 1.27 million deaths were directly attributable to bacterial AMR, with 4.95 million deaths associated with resistant infections across 204 countries and territories [16]. This crisis is further compounded by the misuse and overuse of antibiotics, with up to 50% of all antibiotics prescribed worldwide deemed unnecessary [17]. Within this broader context, the oral cavity has been recognized as a significant site for both the study and dissemination of resistance [10,18]. Dentistry represents an important contributor to antibiotic use, with substantial antibiotic prescribing by dental professionals documented in the United States [19]. Importantly, dental antibiotic prescribing varies geographically and across dental specialties; although overall prescribing rates remained relatively stable between 2012 and 2019, increases were observed in the Northeast and among several dental specialties [19]. Such variability may reflect differences in prescribing practices, clinical guidelines, antimicrobial stewardship policies, and healthcare systems, resulting in differences in antibiotic exposure and selective pressure within oral microbial communities. These considerations are particularly relevant when interpreting geographic differences in oral antimicrobial resistance profiles and resistomes. Beyond the prescription of antibiotics, there is growing concern regarding the widespread use of over-the-counter antiseptics and biocides, such as chlorhexidine digluconate and cetylpyridinium chloride, which may also contribute to the co-selection of antibiotic resistance and persistence of resistant microbial populations within the oral cavity [18,20].

### 3.2. Oral Dysbiosis, Systemic Health, and Dissemination of Antimicrobial Resistance

The oral microbiome exists in dynamic equilibrium with the host, and disturbances in this homeostasis, termed dysbiosis, drive deleterious shifts in microbial composition and metabolic activity, with consequences extending to systemic health [8,21,22]. These shifts can precede clinical signs, involving subtle changes in composition, metabolism and gene expression without major changes in total microbial biomass [23].

Emerging evidence demonstrates clear associations between oral dysbiosis and a spectrum of systemic conditions, including cardiovascular disease, type 2 diabetes mellitus, chronic respiratory infections, and adverse pregnancy outcomes, neurodegenerative disorders, chronic kidney disease, autoimmune diseases, and cancer [8,24,25]. The proposed mechanisms underpinning these associations are multifaceted, encompassing transient or persistent bacteraemia, systemic inflammation caused by microbial metabolites and endotoxins, disruption of immune homeostasis, molecular mimicry, and modulation of host metabolic pathways [8]. As oral bacteria enter the bloodstream through routine activities such as tooth brushing, dental procedures, or periodontal disease, they carry with them the potential to transfer ARGs to microbial communities at distant body sites. The detection of ARGs in streptococcal species associated with infective endocarditis further emphasizes the clinical importance of investigating oral bacteria as sources of systemic infections [12]. The dissemination of antimicrobial resistance genes from the oral cavity into the systemic environment thus poses a significant public health risk, potentially compromising the efficacy of antimicrobial treatments for a wide range of systemic conditions and contributing to the broader spread of resistance within and between individuals [9,10,26]. Furthermore, surveillance of oral resistomes is recognized as a key tenet of the One Health approach to combating AMR, given that the oral microbiome represents the second most significant microbial presence within the body after the gut, serving as a critical interface between the body and the external environment [26,27].

## 4. The Oral Resistome: Composition, Dynamics, and Dissemination

### 4.1. Definition and Conceptual Framework of the Oral Resistome

The oral resistome represents a fundamental shift in our understanding of microbial defense mechanisms and AMR, underscoring the oral microbiome’s critical role as a reservoir and transmission hub for resistance genes. Rather than focusing solely on antibiotic resistance in specific pathogenic bacteria, the oral resistome encompasses the entire collection of antimicrobial resistance genes (ARGs) present within the oral microbiome [9,10]. The oral cavity hosts a complex and dynamic microbial ecosystem that plays a central role in both local and systemic homeostasis [8,22]. This collective genetic reservoir includes genes conferring resistance to a broad spectrum of antimicrobial agents, such as antibiotics, biocides, and antiseptics, commonly utilized in dental practice [18,20]. Remarkably, ARGs are present even in individuals who have not been recently exposed to antibiotics, including those from isolated indigenous populations, suggesting that these genetic elements are intrinsic components of the oral microbial ecosystem [28,29].

Borrowing from the concept of pan-genome, in which genes within the same species are classified as core (shared across essentially all members), accessory/dispensable (present in a subset), or strain-specific [30], resistome research has adopted an analogous “core” versus “accessory” classification [31]. The core resistome comprises ARGs consistently detected across the majority of individuals or samples within a given ecological niche, often reflecting deeply embedded, vertically inherited, or intrinsic determinants; the accessory resistome, conversely, encompasses genes present only in a subset of individuals, frequently associated with mobile genetic elements and therefore more prone to horizontal dissemination and more responsive to selective pressures such as antibiotic exposure or diet [31].

### 4.2. Horizontal Gene Transfer and Dissemination of Antimicrobial Resistance

The oral cavity functions as a critical reservoir for ARGs due to the exceptionally high density and diversity of its microbial communities [32]. A wide diversity of microbial taxa are capable of colonizing the oral cavity, organized within biofilms whose characteristics, including close proximity and polymicrobial nature, enable extensive interactions such as horizontal gene transfer (HGT) [33,34]. This unique environment facilitates HGT between commensal bacteria, enabling the exchange of genetic material even in the absence of external selective pressures such as antibiotic administration [10,35]. The oral microbiota not only maintains local tissue homeostasis but also serves as a reservoir for ARGs that can disseminate both locally and systemically, with the oral cavity acting as a hotspot for the exchange of resistance-conferring mobile genetic elements [32,36,37].

Metagenomic investigations have demonstrated that this genetic exchange is an ongoing process, with resistance genes shared among diverse bacterial species through conjugation, transformation, and transduction [35,38]. More recently, a novel mechanism of HGT involving membrane vesicles has been recognized, although it appears to occur less frequently [38]. The membrane vesicles are spherical nanoscale structures that are naturally released by bacteria during growth and can carry small plasmids and chromosomal DNA fragments carrying ARGs [39]. Upon fusion with the membrane of recipient cells, those membrane vesicles deliver their genetic cargo into the cytoplasm, where the transferred ARGs may be incorporated into the recipient chromosome or maintained as extrachromosomal elements, thereby contributing to the acquisition and dissemination of antimicrobial resistance [39]. Soler and Forterre [40] proposed the term ‘Vesiduction’ for this fourth mode of intercellular DNA transfer.

### 4.3. Composition, Distribution, and Determinants of the Oral Resistome

The composition of the oral resistome has been investigated across different anatomical sites and populations. A systematic review mapping the oral resistome identified 158 unique ARGs across six oral cavity locations, including supragingival and subgingival biofilm, mucosa, oropharynx, root canal system, and saliva, with tetracycline resistance genes dominating across all sites [9]. Three core ARGs, tet(M), tet(O), and ermB, were found universally across all niches regardless of health status, underscoring the stable and inherent nature of this resistance reservoir and suggesting the presence of a core oral resistome [9].

The oral resistome is also established early in life and may undergo changes in its mobilization potential over time. A longitudinal pediatric study identified 309 ARGs from 530 oral metagenomes across the first decade of life, confirming that ARGs form a resilient and stable community within the oral microbiome from infancy, with increasing potential for mobilization as children age [26]. Furthermore, the same study confirmed that co-location of ARGs and insertion sequences has been identified in 27 oral species, confirming the increased HGT potential of oral biofilms [26].

The distribution of ARGs also varies according to antimicrobial class and the characteristics of the population studied. A comprehensive shotgun metagenomic study of 179 individuals identified 64 ARGs conveying resistance to 36 antibiotics, particularly tetracycline, macrolide-lincosamide-streptogramin, and beta-lactam classes, across healthy, caries-active, and periodontally diseased individuals [32]. Moreover, the study by Tansirichaiya et al. [41] demonstrated that healthy individuals from Thailand and Norway harbor distinct oral resistomes despite broadly similar oral microbiomes. Thai participants carried a significantly more diverse repertoire of ARGs, driven by higher abundances of multi-biocide, nucleoside, and copper resistance genes, whereas Norwegian participants exhibited enrichment in aminoglycoside, sulfonamide, and quaternary ammonium compound resistance genes. The authors concluded that geography, antimicrobial exposure, and societal factors shape the oral resistome more strongly than microbial community composition, supporting the inclusion of the oral cavity as a target for antimicrobial resistance surveillance within a One Health framework [41].

### 4.4. Oral Health, Dysbiosis, and Resistome Variation

The presence of ARGs in healthy individuals indicates that antimicrobial resistance is not exclusively associated with disease but constitutes a natural component of the oral microbial ecosystem [9,32,41]. However, the composition and abundance of the oral resistome may vary according to the health status of the oral cavity, although current evidence remains inconsistent regarding the extent and direction of these differences.

Several studies have therefore investigated whether dysbiosis and oral diseases, particularly periodontitis and peri-implantitis, are associated with distinct resistome profiles compared with healthy conditions. The study of Kang et al. [42] showed an increased number of ARGs and significant alterations in the composition of ARGs in dental plaque associated with periodontitis compared to the healthy condition. In contrast, other studies reported a higher prevalence of ARGs in healthy and caries-active individuals than in periodontally diseased individuals [32]. Furthermore, in the study by Bessa et al. [43] that aimed to explore the prevalence and distribution of ARGs in metagenomes derived from saliva and subgingival peri-implant biofilms, they found no significant differences in ARG diversity between healthy and peri-implantitis-affected subgingival biofilm groups.

Geographic variation may further contribute to differences in resistome profiles among individuals with similar or different periodontal conditions. A recent study [44] characterizing the subgingival resistome of healthy subjects (HS) and periodontitis patients (PP) from Belgium, Chile, Peru, and Spain, found that ARG richness was significantly higher in PP than in HS. Moreover, PP from Peru harboured a greater diversity of ARGs than HS from Chile and Spain, highlighting that subgingival resistome profiles vary substantially according to both periodontal status and geographic origin [44].

The heterogeneity among these findings may partly reflect differences in study design and methodological approaches, including sampling site, population characteristics, recent antimicrobial exposure, sequencing depth, ARG databases and bioinformatic pipelines, and normalization strategies. These methodological considerations, and their implications for resistome characterization, are further addressed in subsequent sections of the manuscript. Differences in the anatomical niches sampled and in the characteristics of the study populations may also influence the composition and abundance of detected ARGs, while variations in analytical sensitivity and database coverage may affect resistome profiles and estimates of ARG richness. At the same time, these methodological differences may coexist with genuine biological variation arising from differences in microbial ecology, disease status, geographic origin, and environmental or societal exposures.

Taken together, these findings indicate that the oral resistome is a dynamic component of the oral microbiome that may be influenced by microbial ecology, oral health status, geographic origin, antimicrobial exposure, and other environmental or societal factors. However, the inconsistent associations between disease and ARG abundance emphasize the complexity of the factors shaping the oral resistome. Comprehensive and continuous characterization is therefore essential to identify prevalent ARGs, assess their mobilization potential, and elucidate the mechanisms underlying their transfer within and beyond the oral environment.

## 5. Metagenomic Approaches for Oral Resistome Characterization

The advent of metagenomic sequencing technologies has fundamentally transformed the study of the oral resistome by enabling comprehensive identification and mapping of resistance genes across the entire microbiome, circumventing the limitations of traditional culture-based methods [10,45]. Metagenomics enables the culture-independent analysis of genomic data from environmental and biological samples, allowing the reconstruction of uncultivable microbial genomes and the large-scale identification and characterization of ARGs within microbial communities [45].

### 5.1. Amplicon Sequencing and Its Limitations for Resistome Studies

16S rRNA amplicon sequencing has been foundational in characterizing the core oral microbiome [5,46,47,48], an approach that continues to underpin recent large-scale characterizations [49,50,51]. However, it targets taxonomic composition rather than functional genes, rendering it inherently unable to detect ARGs directly [52]. While it can identify taxa potentially associated with resistance, it cannot reveal ARG diversity, abundance, or mobility potential. Complete metagenomic sequencing, by contrast, enables simultaneous study of structural and functional community diversity, including gene and pathway identification and near-complete genome reconstruction, making it essential for resistome investigations [45,53].

### 5.2. Shotgun Metagenomics

Shotgun metagenomic sequencing has established itself as a particularly informative approach to the study of the oral resistome, as it allows for a comprehensive analysis of the DNA present in a sample, without the need for prior culture or amplification of target genes. As a non-targeted approach, shotgun metagenomics identifies all genes in all organisms without prior knowledge of target sequences [54]. However, the quality of the results obtained depends heavily on the experimental and bioinformatic workflow adopted. This comprises a series of sequential steps, including sample collection, microbial DNA extraction, preparation of sequencing libraries, high-throughput sequencing and the subsequent bioinformatic analysis, which is responsible for the identification and annotation of ARGs [55,56]. Each of these steps can influence the sensitivity, resolution and reliability of the analysis, thereby affecting the characterisation and final interpretation of the resistome [57].

In the context of the oral cavity, the selection of the sampling site is particularly crucial, as the different oral niches, namely saliva, supragingival and subgingival biofilms, the oral mucosa, the oropharynx and the root canal system, harbour distinct microbial communities and exhibit specific antibiotic resistance profiles [9]. Following collection, total DNA is extracted using protocols that ensure the recovery of high-quality genetic material representative of the microbial community present in the sample [56]. In oral samples, the high proportion of host DNA can compromise sequencing depth and reduce the sensitivity of detection of microorganisms and resistance genes. For this reason, strategies for human DNA depletion or microbial DNA enrichment are frequently employed, with the aim of maximising the fraction of microbial DNA available for sequencing and, consequently, improving the quality and sensitivity of metagenomic analyses [58,59].

The next stage in the shotgun metagenomic sequencing workflow involves the preparation of sequencing libraries and the acquisition of data via high-throughput sequencing. Library preparation involves fragmenting the previously extracted DNA and ligating specific adapters to the ends of the resulting fragments [56]. These adapters enable the DNA molecules to be recognised and captured by the various high-throughput sequencing platforms available, which fall broadly into two categories: short-read platforms, such as Illumina NGS, which generate reads typically ranging from 50 to 300 bp; and long-read platforms, such as Oxford Nanopore Technologies and Pacific Biosciences [56].

Following sequencing, the data generated undergo a series of bioinformatic analyses designed to transform the raw sequencing data into biologically interpretable information. The initial stages generally involve data quality control, including the assessment of parameters such as read quality and the removal of low-quality sequences or potential contaminants [56,60]. Subsequently, the sequences obtained are compared with reference databases, which play a central role in characterising the resistome, as they bring together information on previously identified and annotated ARGs [61].

According to Inda-Díaz et al. [62], only a small fraction of the total pool of ARGs detectable across bacterial populations is currently represented in existing resistance gene databases. These are termed established ARGs and typically have been identified in clinical pathogens and are therefore well characterized. In contrast, the vast majority of ARGs, latent ARGs, remain poorly studied [62]. To address this knowledge gap, recent computational approaches have been developed to systematically identify and characterize these latent ARGs within bacterial genomes [63,64,65,66]. Applied to the oral cavity, this framework allows researchers to distinguish resistance genes that are a stable, near-universal feature of the “healthy” oral resistome from those that are more idiosyncratic, environmentally acquired, or disease-associated, offering a route toward identifying which elements of the resistome are most informative for risk stratification.

#### 5.2.1. Long-Read Sequencing Technologies

A fundamental limitation of short-read metagenomics is the inability to confirm associations between ARGs, mobile genetic elements (MGEs), and host species, as reads spanning only 150–300 bp cannot resolve complex genomic contexts [67,68]. Third-generation sequencing technologies, also known as long-read sequencing technologies, overcome this limitation by continuously sequencing DNA fragments several kilobases to tens of thousands of base pairs in length, providing a more comprehensive view of the genetic context. Among these, Oxford Nanopore Technologies (ONT) and Pacific Biosciences (PacBio) are the two main platforms used in metagenomic studies, enabling improved genome assembly, resolution of complete plasmid and transposon structures, and direct linkage of ARGs to their host species [69,70,71,72].

ONT enables the direct real-time sequencing of DNA fragments as they pass through nanopores embedded in a membrane. This technology is capable of generating ultra-long reads, facilitating the reconstruction of bacterial genomes and the characterisation of the genetic context of ARGs [69]. Furthermore, the availability of small, portable devices, such as the MinION, enables sequencing to be carried out in a variety of settings, facilitating applications in microbiological and epidemiological surveillance [73,74,75]. Although historically associated with lower accuracy per read, recent advances in sequencing chemistry and error-correction algorithms have significantly improved its performance, making ONT an increasingly widely used tool in metagenomic studies of the resistome [76,77,78].

The PacBio platform is based on Single Molecule Real-Time (SMRT) technology, which enables the sequencing of individual DNA molecules in real time, without the need for prior amplification [69,79]. With the development of HiFi (High-Fidelity) technology, PacBio has been able to generate long reads with high accuracy, enabling the reconstruction of high-quality microbial genomes and a more reliable identification of ARGs, as well as their genomic context, including mobile genetic elements [80,81].

Target-enriched long-read sequencing (TELSeq) further enhances sensitivity by selectively enriching ARG-containing DNA fragments prior to sequencing while preserving their surrounding genomic context, enabling characterization of adjacent mobile genetic elements and other flanking genes [82]. Fuhrmeister et al. [83] introduced Context-Seq, a CRISPR-Cas9-targeted long-read sequencing method for ARGs and their genomic context, used to investigate genetically similar antimicrobial resistance elements shared among adults, children, poultry, and dogs in Kenya, identifying genetically distinct clusters shared between humans and animals within and between households. Although developed and validated in non-oral contexts, technologies such as Context-Seq [83] and long-read metagenomic profiling of animal resistomes [84] illustrate the potential of targeted and long-read approaches to resolve ARG genomic context, mobility, and host associations. These findings provide a methodological basis for considering similar approaches in oral resistome research; however, their ability to resolve ARG–host associations and transmission pathways in oral microbial communities remains to be directly demonstrated.

While direct applications of long-read sequencing to oral resistome characterization remain limited, emerging evidence confirms their feasibility and particular value in this context. Baker [85] demonstrated that combined ONT and Illumina sequencing can recover eleven complete bacterial genomes from human saliva metagenomes, establishing proof-of-concept for full-resolution genome assembly from this oral matrix, an essential prerequisite for placing ARGs in their complete chromosomal or plasmid context. Comparative studies have further shown that full-gene 16S rRNA ONT sequencing achieves markedly superior species-level resolution for salivary microbiome profiling compared with Illumina MiSeq [86], and PacBio HiFi sequencing has been applied to characterize oral microbiome composition across diverse human populations, identifying 236 genera and 376 species and revealing significant population-specific community structures [87]. Although these studies do not directly characterize the oral resistome, they demonstrate the applicability of long-read technologies to oral samples and support their potential utility for future resistome investigations. In the specific context of the oral resistome, Sukumar et al. [9] explicitly identified long-read metagenomic platforms as state-of-the-art tools for establishing ARG–species associations, with the capacity to reveal effects of AMR on bacterial physiology that remain inaccessible to short-read approaches.

Given that the confirmed physical linkage of ARGs to their MGE and host-species contexts is recognized as a critical methodological gap in current oral resistome studies [15,41], the integration of long-read sequencing into future investigations, particularly those involving salivary metagenomes and subgingival biofilm samples, may help address this gap. However, further studies directly applying these approaches to oral resistome characterization are needed to establish their performance, sensitivity, and ability to resolve ARG–MGE–host relationships and transmission pathways in oral microbial communities.

#### 5.2.2. Shotgun Metagenomics Versus Targeted, PCR-Based Approaches

The principal advantage of shotgun metagenomics over targeted, PCR-based approaches lies in its capacity for comprehensive, unbiased detection of the resistome. Because it sequences all genomic DNA present in a sample rather than amplifying a predefined set of targets, shotgun metagenomics substantially outperforms PCR in both the breadth and depth of ARG detection. A systematic review of the oral resistome found that next-generation sequencing (NGS)-based metagenomic studies identified an average of 34 ARGs and 177 ARG-carrying species per study, compared to only 7 ARGs and 25 species detected through PCR-based methods [9]. This order-of-magnitude difference illustrates the central advantage of a non-targeted approach: the ability to capture the full diversity of resistance determinants and their bacterial hosts without prior assumptions about which genes are present.

This capability has proven particularly valuable for large-scale, longitudinal characterisation of the oral resistome. Sukumar et al. [26] applied shotgun metagenomics to 530 oral samples collected from 221 twin children across the first decade of life, identifying 309 distinct ARGs and demonstrating that resistome composition clusters significantly by age, with detectable host genetic effects present from infancy onwards. Such findings would be unattainable using targeted amplicon or PCR-based methods, which are inherently constrained to previously characterised sequences and cannot reveal age-related shifts in the overall resistome landscape or the emergence of novel resistance determinants.

Beyond gene discovery, shotgun metagenomics has also been instrumental in revealing discrepancies between genotypic and phenotypic resistance profiles, underscoring a key limitation of relying on sequencing data alone. In a large-scale study of 179 individuals, ref. [32] combined shotgun metagenomic sequencing with culture-based phenotypic testing for the first time in an oral biofilm context, and observed a clear mismatch between the resistance genes detected genomically and the resistance phenotypes observed experimentally. This finding highlights an important challenge inherent to metagenomic approaches: the mere presence of an ARG sequence does not guarantee its expression or functional relevance, and genomic prediction alone may overestimate or underestimate the true resistance phenotype of a microbial community. Anderson et al. [32] therefore emphasised the necessity of combining multiple complementary methods, rather than relying on shotgun metagenomics in isolation, to accurately characterise antibiotic resistance within complex oral biofilms. This gap between genotype and phenotype is precisely where sequence-based functional metagenomics becomes indispensable: by directly linking genomic sequence data to functional resistance phenotypes, it offers a powerful means of discovering novel resistance genes and elucidating resistance mechanisms that would otherwise remain invisible to conventional microbiological and purely sequence-based approaches alike [45].

### 5.3. Functional Resistome vs. Genetic Resistome

The resistome can be characterised through two complementary methodological lenses: the genetic (or sequence-based) resistome and the functional resistome, and this distinction has become especially prominent in recent microbiome literature as sequencing costs have fallen and functional screening platforms have matured (Table 1).

The genetic resistome is typically inferred by aligning shotgun metagenomic sequencing reads against curated ARG reference databases such as CARD, ResFinder, MEGARes, NCBI AMRFinderPlus or ARG-ANNOT, a strategy that is fast and highly scalable, making it well suited to routine surveillance, but one whose sensitivity is intrinsically bounded by the completeness of these databases [88]. Because such databases are built predominantly from ARGs characterized in culturable, clinically relevant pathogens, this homology-based approach is systematically biased against resistance determinants from non-culturable or non-pathogenic taxa and against genes that are highly divergent from previously described sequences, limiting its capacity to detect truly novel ARGs [89]. More recent work extending this logic to a clinical oral setting has used shotgun metagenomic sequencing data of dental plaque to characterise ARG profiles in patients before and after scaling and root planing therapy, observing a significant shift in the resistome post-therapy with important clinical implications [42]. Building on this, subsequent comparative metagenomic analyses have further mapped the subgingival resistome and its associated mobile genetic elements across health and periodontitis, reinforcing the link between dysbiosis, treatment response, and resistance gene dynamics [44]. Moreover, methodological factors upstream of sequencing itself can further shape what the genetic resistome reveals. For instance, a 2024 study comparing DNA extraction protocols from saliva samples found that a MetaPolyzyme chemical cell-lysis treatment led to significant shifts in microbial composition, favoring Gram-positive bacteria such as *Streptococcus* over Gram-negative counterparts, and produced a distinct change in ARG distribution characterized by an elevated proportion of fluoroquinolone- and efflux-pump-associated genes alongside a reduction in tetracycline and β-lactam resistance genes compared with untreated samples, underscoring that the genetic resistome reported by a given study is partly an artefact of laboratory workflow rather than a fixed biological property of the sample [90].

The functional resistome, by contrast, is defined experimentally using functional metagenomics: metagenomic DNA fragments are shotgun-cloned into heterologous hosts (typically *Escherichia coli*) and challenged with antimicrobial agents, thereby enabling resistance to be identified directly from observed phenotypes rather than inferred from sequence similarity [91,92]. The DNA fragments responsible for the resistant phenotype are then sequenced and annotated. This approach is particularly valuable in the oral cavity, where a substantial proportion of resident bacteria remain difficult or impossible to culture by conventional means. A functional metagenomic screen of an oral metagenomic library constructed from saliva samples of 50 healthy Norwegian volunteers identified resistance determinants to chlorhexidine and sodium hypochlorite, several of which showed no meaningful similarity to previously characterised resistance genes and would therefore have been missed by sequence-based approaches alone [93]. Newer hybrid strategies, such as bacteriophage-assisted multi-species functional metagenomics, have sought to expand the range of indicator hosts and thereby capture functional resistomes closer to their native genomic and taxonomic context [94]; however, to the best of our knowledge, they have not been applied to the study of the oral resistome.

### 5.4. Bioinformatics and Computational Tools

#### 5.4.1. ARG Databases

Appropriate database selection critically determines the scope, sensitivity, and interpretability of resistome analyses, since ARG annotation is fundamentally constrained by the breadth, curation quality, and update frequency of the reference database against which sequencing reads or assembled contigs are compared [61].

Among the databases currently available, the Comprehensive Antibiotic Resistance Database (CARD) is the most widely adopted in metagenomic projects, providing manually curated gene and protein entries that combine the Antibiotic Resistance Ontology (ARO) with reference AMR sequences and resistance-conferring mutations, covering both intrinsic and acquired resistance mechanisms [95]. Its accompanying Resistance Gene Identifier (RGI) software further allows direct annotation of genomic and metagenomic sequences against these curated models, making CARD not merely a static repository but an integrated analytical framework for resistome interpretation. Since its original release, CARD has expanded substantially: the 2023 update added 180 new AMR gene families and 15 drug classes, extended in silico resistome prediction to 377 pathogens and hundreds of thousands of genomic assemblies, and introduced standardised gene identifiers to support machine learning applications [96]. This reflects a broader shift from static reference libraries toward predictive, structured resistome resources, an evolution particularly relevant for oral metagenomic and metatranscriptomic studies, where reliable ARG detection and downstream inference about mobility, host association, or expression depend directly on database completeness and currency.

Beyond CARD, several other curated databases are routinely used for ARG annotation, each with distinct scope, curation philosophy, and update cadence, factors that Papp and Solymosi [61] systematically compared in their review of antimicrobial resistance gene databases. ResFinder, maintained by the Center for Genomic Epidemiology, focuses specifically on horizontally acquired resistance genes and associated point mutations, and is widely used for phenotype prediction directly from genomic data [97]. MEGARes, by contrast, was purpose-built for high-throughput metagenomic analysis, offering a hierarchical annotation structure that extends beyond antibiotic resistance to include biocide and metal resistance determinants, and is distributed alongside the AMR++ bioinformatic pipeline for streamlined classification of sequencing reads [98]. The NCBI Reference Gene Catalog, accessed through the AMRFinderPlus tool, represents a further widely used option, distinguished by its integration with NCBI’s broader genomic infrastructure and its inclusion of stress-response and virulence genes alongside canonical AMR determinants [99].

Papp and Solymosi [61] demonstrated that these databases differ substantially not only in the number of genes and families represented, but also in curation criteria, sequence redundancy, and the frequency and transparency of their update cycles, differences with direct downstream consequences for resistome sensitivity and reproducibility. Critically, the same set of sequencing reads or assembled contigs annotated against different databases can yield materially different resistome profiles, underscoring that database choice is not a neutral methodological step but an active determinant of the biological conclusions drawn from any given oral, or other, metagenomic resistome study.

#### 5.4.2. Analytical Pipelines and Machine Learning

Standardised bioinformatic pipelines integrate several complementary tools to move from raw sequencing reads to a fully annotated resistome. Quality filtering and host-read removal are typically performed with KneadData, part of the bioBakery suite of metagenomic tools [100], after which cleaned reads are assembled into contigs using metaSPAdes [101] or MEGAHIT [102], the latter being particularly well suited to large, complex metagenomic datasets due to its use of succinct de Bruijn graphs. ARG annotation is then performed against curated databases such as CARD using tools like ABRicate [103], while downstream statistical analysis, including differential abundance testing and taxonomic/functional data visualisation, is commonly conducted in R using packages such as DESeq2 [104] and phyloseq [105]. Together, these tools form an end-to-end pipeline capable of moving from raw sequence data to statistically robust resistome and microbiome comparisons across samples or study groups.

Beyond these established pipelines, machine learning and artificial intelligence represent rapidly emerging tools for resistome research, particularly for overcoming the homology-based limitations inherent to conventional database annotation. Deep learning models such as DeepARG were specifically developed to address the high false-negative rates of traditional best-hit homology searches, using the full similarity distribution of a query sequence against known ARGs, rather than relying on a single best match, allowing the identification of divergent or previously uncharacterised resistance genes that would otherwise be missed by standard annotation pipelines [106]. Beyond gene-level prediction, machine learning approaches have also been applied to classify resistance phenotypes directly from omics data: for example, proteomic data generated by mass spectrometry has been combined with CARD-derived reference information to detect antimicrobial resistance directly at the protein level, illustrating how machine learning can bridge genomic, proteomic, and phenotypic layers of resistome characterisation [107]. As these approaches mature, they are expected to complement, rather than replace, curated database annotation, offering a scalable means of detecting novel resistance determinants that fall outside the boundaries of current reference knowledge.

#### 5.4.3. Key Challenges

Despite these advances, several important methodological challenges persist, and they compound one another across the resistome analysis pipeline. Annotation accuracy remains fundamentally database- and pipeline-dependent: a recent large-scale comparison of ten commonly used ARG detection pipelines, applied to over 270 million prokaryotic genes across 13 distinct habitats, found up to a 45-fold difference in the number of ARGs reported and a mean pairwise agreement (Jaccard index) of only 16% between pipelines, with pipeline choice substantially altering downstream estimates of ARG abundance, richness, and pan- and core-resistome composition [108]. This confirms, at unprecedented scale, the earlier observation that generalist databases can introduce niche-specific bias and that no single tool or database should be treated as an authoritative reference standard [61].

Novel ARG detection is further constrained by the limitations of homology-based annotation itself, which shows reduced sensitivity for minority-population and divergent ARGs [106], and often lacks the resolution needed to distinguish specific allelic variants, a distinction with direct consequences for phenotypic interpretation, since not all alleles of a given ARG family confer the same resistance profile, as demonstrated for the aac(6′) aminoglycoside resistance gene family, where sequence-homology-based annotation failed to reliably predict phenotypic susceptibility across clinical isolates [109]. 

Quantification adds another layer of inconsistency: the choice of normalisation method, such as RPK versus TPM, is not a neutral technical decision, since these metrics reflect the relative abundance of a target within the sequenced population rather than an absolute quantity, meaning that values are not directly comparable across samples or studies with differing community composition or sequencing depth [110]. This lack of standardisation in normalisation and abundance metrics remains a major obstacle to meaningful cross-study comparison of resistome data [81].

Host assignment presents an additional, largely unresolved challenge. ARG-to-host linkage typically relies on contig-level taxonomic classification tools such as Kraken2 [111], but genes located on plasmids, transposons, or other mobile genetic elements frequently share sequence features across taxonomically diverse hosts, causing them to remain unclassified or to be assigned only to the root of the taxonomic tree rather than to a specific organism [112]. These limitations highlight the urgent need for standardised bioinformatic pipelines, unified normalisation and reporting parameters, and, where possible, transcriptomic or phenotypic validation, to enable reproducible and genuinely comparable oral resistome research. Given the substantial influence of analytical choices on resistome characterization, the major sources of variability and their potential consequences are summarized in Table 2.

## 6. Multi-Omics Integration in the Oral Resistome Study

Multi-omics integration extends resistome characterisation beyond what DNA-based detection alone can offer, enabling the different biological layers, genomic, transcriptomic, proteomic, and metabolomic, to be analysed jointly rather than in isolation. One framework increasingly used to achieve this integration is DIABLO (Data Integration Analysis for Biomarker discovery using Latent cOmponents), a sparse Partial Least Squares Discriminant Analysis (sPLS-DA)-based method that simultaneously identifies correlated patterns across ARG profiles, taxonomic composition, and functional pathway data [113]. By modelling these relationships jointly, such multi-omics approaches can reveal mechanistic interactions that remain invisible to single-omics strategies applied independently, offering a substantially more complete picture of oral resistome ecology [113,114].

### 6.1. Metatranscriptomics

Metatranscriptomics captures actively expressed ARGs, distinguishing genes present in the metagenome from those actually transcribed; this layer is necessary to fully elucidate the relationship between resistance genes and bacterial metabolism [115].

The application of metatranscriptomics to oral microbial communities remains comparatively limited relative to metagenomics, but the studies conducted to date have consistently demonstrated that transcriptional activity diverges substantially from taxonomic or genomic abundance profiles. Belstrøm et al. [116] applied paired metagenomic and metatranscriptomic sequencing to subgingival plaque, tongue, and saliva samples from periodontitis patients and healthy controls, quantifying species-specific bacterial activity as the ratio of mRNA reads to corresponding genomic DNA reads. Their findings revealed that periodontitis was associated with a significant reduction in carbohydrate metabolism-related gene expression across all three oral sites, even though oral site, rather than disease status, remained the primary determinant of overall taxonomic composition. This dissociation between “who is present” and “what is being expressed” illustrates a broader principle directly relevant to resistome analysis: the same logic applies to ARGs, as their genomic presence alone cannot establish whether they are biologically active under a given physiological or pathological condition. Similarly, Pinheiro et al. [117] conducted, to the best of current knowledge, the first combined metatranscriptomic and resistome analysis of the endodontic microbiome, directly characterising the transcriptional activity of ARGs within root canal infections rather than relying solely on their genomic detection.

More broadly, Huang et al. [118] reviewed the combined use of metagenomics and metatranscriptomics in periodontitis research, emphasising that functional activity profiling is essential to bridge the gap between microbial dysbiosis, observed at the compositional level, and the pathogenic mechanisms driving disease progression. The authors highlight that overrepresentation of genes related to antibiotic resistance, adhesion, and stress response at the DNA level does not necessarily equate to their transcriptional activation, reinforcing the need for RNA-level validation.

Metatranscriptomics, though still underutilised in oral microbiome research compared to shotgun metagenomics, represents a critical complementary layer for resistome characterization. Its integration with genomic and, where possible, metaproteomic data offers a more complete picture of the functional dynamics of antimicrobial resistance in the oral cavity, one capable of distinguishing genetic potential from realised phenotype, and thereby better informing the clinical risk posed by the oral resistome.

### 6.2. Metaproteomics and Metabolomics

Metaproteomics and metabolomics provide complementary layers of functional resolution for resistome characterisation. Metaproteomics confirms the functional translation of resistance determinants at the protein level, verifying that a transcribed ARG is not only present as mRNA but is actually translated into a functional protein product, while metabolomics reveals the biochemical context in which resistance is maintained, linking resistance gene activity to the metabolic state of the surrounding microbial community. In principle, detecting a resistance gene in the metagenome, or even its transcript in the metatranscriptome, is not sufficient to establish that the encoded protein is produced and functionally active; this distinction requires direct protein-level detection and, ideally, phenotypic validation [119]. In oral biofilms, the metabolic dimension of this multi-omics picture already has direct empirical support, even though the proteomic confirmation of ARG translation specifically remains largely unexplored.

Complementary evidence from oral metaproteomic studies supports the broader principle that sugar metabolism reshapes the functional protein landscape of oral biofilms in ways relevant to dysbiosis, even though these studies have not yet targeted ARGs specifically [120,121]. Rudney et al. [121], using a paired oral microcosm biofilm model of dental caries, demonstrated that sucrose exposure induced consistent shifts in protein relative abundance across taxonomically diverse communities, particularly in pathways involving glycolysis, lactate production, aciduricity, and ammonia/glutamate metabolism, changes that were conserved across individuals despite considerable inter-subject taxonomic variability. This indicates that function-based protein signatures, rather than taxonomic composition alone, may serve as more reliable biomarkers of the metabolic shifts that accompany dysbiosis, an approach directly extendable, in principle, to tracking the functional expression of ARGs and resistance-associated proteins under similar sugar-rich, low-pH conditions.

Whether this extension has actually been made, however, remains an open question. A recent scoping review of the subgingival resistome in periodontitis noted that, although numerous studies have characterised rich repertoires of tetracycline, macrolide, and β-lactam ARGs, none of the available studies had assessed ARG expression at the RNA or, especially, the protein level, explicitly identifying metaproteomic and functional validation studies as essential future priorities to discriminate “silent” genetic resistance potential from resistance that is clinically and metabolically meaningful [15].

The oral resistome is not a metabolically inert genetic reservoir, but one whose diversity and persistence appear entangled with the biofilm’s carbohydrate metabolism. Confirming this relationship directly at the protein and metabolite levels, rather than inferring it solely from the parallel patterns observed in genomic and general metaproteomic studies, remains an important direction for future oral multi-omics research, particularly given that resistance gene diversity appears to co-occur with, and may be sustained by, the same metabolic pathways that drive oral biofilm formation.

## 7. Precision Oral Health and the Future of Personalized Dentistry

The integration of oral microbiome and resistome analysis is central to the emerging paradigm of precision oral health and personalised dentistry. Precision medicine develops targeted therapies for individual patients by integrating data across multiple omics layers, genomics, transcriptomics, epigenomics, metabolomics, and proteomics, to build predictive models of individual biological systems [13]. Within the oral cavity, this concept, termed “precision dentistry,” reframes the prevention and treatment of caries, periodontitis, oral cancers, and related systemic conditions around the individual patient rather than population-level guidelines, leveraging tools such as salivary biomarkers, point-of-care diagnostics, and genomic data to support individualised treatment strategies [13,122,123]. In this context, the potential contribution of resistome profiling to precision oral health can be considered from three complementary perspectives: the biological and ecological determinants underlying individual resistome profiles, the potential clinical and translational applications of resistome-informed approaches, and the methodological, infrastructural, ethical, and evidence-related barriers that currently limit their clinical implementation.

### 7.1. From Resistome Profiling to Precision Oral Health

Any precision-oriented interpretation of an individual’s resistome must first account for the fact that it is not a static entity, but rather a dynamic component of the oral ecosystem that begins to take shape from the earliest stages of life [26]. As introduced in Section 4.1, ARGs are already detectable in newborns, with resistome diversity increasing steadily during the first two years of life before stabilising by approximately five years of age [26]. This early trajectory appears to be shaped jointly by host genetic background and environmental exposure. Evidence for a heritable component to the oral microbiome more broadly is mixed: twin studies report greater similarity between monozygotic than dizygotic twins for specific taxa, while others suggest environmental factors predominate [124], and a separate large-scale metagenome-wide association study of over 1915 individuals found that host genetics accounts for at least 10% of the variance in overall oral microbiome composition [125]. While this genetic signal has been established for the oral microbiome rather than the resistome specifically, it is consistent with, and lends plausibility to, the detectable host genetic effects on ARG diversity that Sukumar et al. [26] observed in the oral resistome from infancy onwards. Feeding practices during infancy appear to exert a measurable influence on this trajectory: exclusively breastfed infants (up to six months) show a greater abundance of resistance genes such as mefA and msrD compared with formula-fed infants [26], suggesting that early dietary exposures modulate not only microbiome composition but also its associated resistance gene repertoire.

This developmental trajectory does not simply plateau into a fixed adult profile; the resistome continues to reorganise in response to disease state throughout life. Certain ARGs act as specific disease markers; for instance, the genes tetA(46) and tetB(46) have been detected exclusively in individuals with active caries, indicating that the resistome undergoes quantitative reorganisation in response to local disease processes rather than remaining a fixed background feature of the oral microbiome [32]. Beyond these individual-level determinants, the mobility of resistance genes and the broader exposure context in which a patient lives further shape how the resistome manifests. The oral cavity functions as a hotspot for horizontal gene transfer, largely facilitated by the Tn916/Tn1545 family of conjugative transposons [126,127]. This mobility underlies co-selection phenomena, whereby exposure to one antimicrobial agent can indirectly select for resistance to another; erythromycin use, for example, has been shown to co-select for tetracycline-resistant bacteria via shared genetic linkage on Tn1545-like conjugative elements [128]. Importantly, as demonstrated in Section 3.1, the geographic divergence observed between Thai and Norwegian resistomes [41] and across the four-country subgingival comparison spanning Peru, Chile, Belgium, and Spain [44] illustrates precisely why population-level exposure history, and not merely host genetics and local microbial ecology, must inform any precision-oriented interpretation of an individual’s resistome profile.

These observations support the concept that precision oral health should consider the resistome as a dynamic phenotype emerging from interactions among host characteristics, microbial ecology, disease status, antimicrobial exposure, diet, geography, and other environmental factors. Such variation provides a biological rationale for moving beyond population-level descriptions of antimicrobial resistance; however, the existence of individual-level variation does not, by itself, establish that resistome profiling can accurately predict clinical outcomes or guide treatment decisions.

### 7.2. Clinical and Translational Potential of the Oral Resistome

Within a precision oral health framework, resistome profiling could eventually complement conventional clinical, microbiological, and patient-level information to refine individual risk assessment and guide preventive or therapeutic strategies. Rather than serving as a stand-alone diagnostic measure, resistome information may be most informative when integrated with clinical findings, oral microbiome composition, antimicrobial exposure history, and other relevant host and environmental factors. Such integration could, in principle, help identify patients or microbial communities with a higher burden of clinically relevant resistance determinants and support more targeted antimicrobial decision-making. However, this framework remains prospective, as the clinical thresholds, predictive validity, and patient outcomes associated with resistome-informed decision-making have not yet been established.

The potential clinical relevance of such approaches must also be considered in light of the relationship between resistance determinants and their functional expression. As outlined in Section 5.2, there is a recurring discrepancy between genotypic resistance detected via shotgun metagenomic sequencing and phenotypic resistance observed in culture, as observed in the study by Anderson et al. [32]. Consequently, detection of an ARG should not automatically be interpreted as evidence that a patient will exhibit a clinically relevant resistant phenotype. Integrating resistome data with phenotypic susceptibility testing, clinical characteristics, and other microbiological measurements may therefore be necessary before such information can reliably inform antimicrobial selection.

Precision oral health should also not be conceptualised solely as the individualised selection of antimicrobial agents. It may encompass strategies aimed at modifying the oral ecological environment and reducing the need for antimicrobial exposure. Emerging adjuncts illustrate this broader ecological perspective. Dietary supplementation with omega-3 fatty acids has been shown to raise salivary Resolvin E1 levels and improve periodontal clinical parameters in patients with chronic periodontitis [129], supporting its potential as a personalised, nutrition-based adjunct in precision periodontal care. Similarly, probiotic strains capable of producing ammonia via the arginine deiminase system (ADS) help neutralise acidic biofilm pH, offering a targeted, patient-specific approach to caries prevention [130]. Together with non-antibiotic strategies such as photodynamic therapy [131], these adjuncts illustrate how precision approaches to oral health are evolving toward a broader ecological management of the oral microbiome, rather than a purely antimicrobial-centred model.

From this perspective, a future resistome-informed precision oral health framework could conceptually progress from individual patient characteristics and exposure history to characterisation of the oral microbiome and resistome, followed by integrated risk interpretation, selection of preventive or therapeutic interventions, and longitudinal monitoring. Such a framework could potentially support more targeted antimicrobial stewardship while also identifying opportunities for non-antibiotic ecological interventions. Nevertheless, this represents a proposed translational model rather than an established clinical workflow. To date, the evidence considered in this review does not establish that incorporation of resistome information into dental decision-making improves patient outcomes or reduces inappropriate antimicrobial prescribing. Interventional studies and clinical trials are therefore needed to determine whether resistome-informed approaches provide measurable clinical benefits and can be implemented reliably in routine dental practice.

### 7.3. Barriers, Evidence Gaps, and Future Directions

Realising the potential clinical value of oral resistome profiling requires resolving persistent methodological limitations. As outlined in Section 5.2, sample-processing choices upstream of sequencing, such as enzymatic cell-lysis treatment with MetaPolyzyme, measurably alter the resistome profile obtained from a given sample, a methodological sensitivity that any precision-based clinical application will need to account for and standardise [90]. More broadly, differences in analytical workflows, reference databases, sequencing approaches, normalization strategies, and approaches to ARG interpretation can affect resistome characterization and complicate comparison between studies. These sources of analytical variability reinforce the need for methodological standardisation before resistome profiles can be reliably translated into clinically actionable information.

Beyond these laboratory-level challenges, routine implementation faces broader infrastructural and ethical barriers. The absence of global data-exchange standards, including limited adoption of terminologies such as SNOMED, continues to fragment patient data across institutions, hindering the aggregation needed for robust predictive modelling [132]. Compounding this, the computational and energy demands of storing and modelling large-scale omics datasets raise questions about the ecological footprint and sustainability of these technologies, which must be weighed against their clinical benefits as adoption scales [132,133]. These constraints are closely tied to ethical concerns over governing sensitive patient data. Collecting and storing genomic, metagenomic, and salivary data raises privacy issues that demand robust informed consent, covering not only immediate clinical use but also future research reuse or third-party sharing [134]. This governance challenge is inseparable from a deeper equity concern: the high costs of AI-based systems and multi-omics data acquisition keep point-of-care diagnostics, sequencing, and AI tools concentrated in higher-resource settings and industrialised nations, since most datasets originate from high-income countries [132,135]. This may disproportionately affect and exclude vulnerable populations, particularly those with lower health literacy or limited access to dental care, from these technologies’ benefits [135]. Equitable access should therefore be as central an objective to precision oral health as the technical advancement of its tools.

A fundamental barrier is the gap between methodological feasibility and demonstrated clinical effectiveness. Although oral microbiome and resistome profiling can provide detailed information on microbial composition and resistance determinants, their clinical relevance, predictive value, and capacity to inform treatment decisions remain incompletely established. Prospective validation is therefore needed to determine whether resistome profiles provide clinically relevant information beyond established parameters and can support antimicrobial stewardship or patient-specific treatment.

Future research should move beyond resistome characterization toward integrated and clinically evaluated approaches combining resistome data with microbiome composition, patient characteristics, antimicrobial exposure, clinical phenotypes, and functional resistance measurements. Prospective cohort studies, standardized multi-centre investigations, and interventional trials will be important to establish clinical utility and determine whether resistome-informed strategies improve outcomes or reduce unnecessary antimicrobial exposure. Implementation should also address interoperability, privacy, sustainability, and equitable access.

Precision oral health thus represents a broader reconceptualisation of individualized risk, diagnosis, prevention, and treatment. The oral resistome may contribute to this framework as a dynamic component of the microbial ecosystem, but its current role remains primarily investigational. Establishing its clinical value will require rigorous validation while maintaining a clear distinction between translational potential and demonstrated clinical benefit.

## 8. Conclusions

The integration of oral resistome data, from its early formation and heritable component, through mobility and geographic modulation, to its links with systemic disease, salivary diagnostics, AI-assisted modelling, and personalised antimicrobial stewardship, outlines a concrete path toward predictive, patient-centred dentistry. Realising this potential depends on resolving the methodological, infrastructural, and ethical barriers discussed above, which should stand as research priorities as the field shifts from a reactive to a preventive, individualised paradigm.

Genetic and functional characterisation of the oral resistome should be regarded as complementary rather than interchangeable. Genetic approaches offer the scalability and throughput needed for population-level surveillance, while functional approaches remain indispensable for validating gene function and discovering novel resistance determinants that sequence-based databases overlook. A purely genomic map risks conflating genetic potential with clinically meaningful resistance; functional approaches alone cannot scale to population-level needs. This complementarity extends to multi-omics and long-read sequencing: long-read platforms link ARGs to their mobile genetic elements and hosts, a prerequisite for functional validation, while metatranscriptomic and metaproteomic layers distinguish a resistome that is merely encoded from one that is actively expressed within the biofilm. The field’s trajectory therefore points toward integrated workflows, combining long- and short-read sequencing, genomic and functional metagenomics, and computational prediction with experimental validation, rather than refinement of any single approach.

Clinical translation, however, is not solely a technical challenge. The methodological inconsistencies across pipelines and databases, the genotype–phenotype discordance observed in oral biofilms, and the geographic and developmental variability of the resistome all underscore the need for standardised, reproducible workflows before resistome data can inform clinical decisions. Likewise, infrastructural and equity concerns, from data-exchange standards to the unequal global distribution of sequencing and AI resources, must be addressed deliberately if the benefits of resistome-informed precision dentistry are to be realised equitably.

Ultimately, the oral resistome should be understood not as a fixed catalogue of resistance genes, but as a dynamic, mobile, and functionally embedded component of the oral ecosystem, one that begins forming in infancy, is shaped by host genetics and environmental exposure, and remains responsive to disease state and antimicrobial pressure throughout life. Fully characterising it, genetically, functionally, and clinically, will require continued convergence of sequencing technology, multi-omics integration, and computational modelling. Achieving this convergence in a way that is methodologically robust, clinically actionable, and equitably accessible represents the central challenge, and opportunity, for the next generation of oral microbiome and antimicrobial resistance research.

## Figures and Tables

**Table 1:** Comparison between Genetic and Functional Resistome characterization.

Methodological Approach	Primary Definition	Detection Mechanism	Strengths and Advantages	Limitations and Biases	Novel ARG Discovery Potential	Clinical Application Scope	Type of Evidence Produced
Genetic (Sequence-based) Resistome	Sequence-based inference defined by alignment against curated ARG reference databases.	Homology-based alignment of shotgun metagenomics against databases (e.g., CARD, ResFinder, MEGARes).	Fast, highly scalable, reproducible, and provides high throughput required for population-level surveillance and large cohorts.	Sensitivity is bounded by database completeness; systematically biased against non-culturable taxa and highly divergent sequences; results can be affected by laboratory workflows.	Limited capacity to detect truly novel ARGs as it depends on similarity to previously described sequences.	Routine surveillance, population-level mapping of ARGs, and assessment of ecological changes post-therapy.	Evidence of the presence and genetic potential of antimicrobial resistance based on the detection of ARG sequences; does not by itself demonstrate that the detected genes are expressed or confer a resistant phenotype.
Functional Resistome	Phenotype-driven screening defined experimentally by identifying resistance based on the ability to survive antimicrobial challenge.	Metagenomic DNA fragments are shotgun-cloned into heterologous hosts (e.g., *E. coli*) and challenged with antimicrobial agents.	Identifies resistance directly from observed phenotype; indispensable for validating gene function and establishing direct genotype-to-phenotype relationships.	Requires heterologous host expression; more labor-intensive and lower throughput than purely computational sequence alignment.	High; capable of discovering genuinely novel resistance determinants that share no similarity with known genes and would be missed by sequence-based methods.	Discovery of novel resistance mechanisms in unculturable taxa, validation of gene function, and informing precision antimicrobial stewardship.	Experimental evidence of functional antimicrobial resistance, demonstrating that genetic material can confer a resistance phenotype under the tested conditions and thereby supporting a direct genotype–phenotype relationship.

**Table 2:** Major sources of analytical variability in oral resistome characterization.

Analytical Factor	Potential Source of Variability	Consequence for Resistome Characterization
ARG database selection	Differences in database scope, curation criteria, sequence redundancy, update frequency, and gene coverage.	Can alter ARG detection sensitivity, richness, and composition, affecting reproducibility and cross-study comparability.
Annotation criteria and thresholds	Differences in sequence identity, alignment coverage, confidence thresholds, and annotation criteria used to assign ARGs.	Can substantially change the number and types of ARGs detected, particularly for divergent or distantly related sequences.
Assembly strategy	Differences between read-based and contig-based approaches, as well as assembly algorithms and parameters.	Influences ARG recovery, genomic-context resolution, and the ability to identify associations with mobile genetic elements (MGEs) or host taxa.
Normalization and abundance metrics	Use of different metrics and normalization strategies, such as RPK, TPM, or other approaches.	Can affect estimates of relative ARG abundance and limit direct comparison across samples and studies.
Host assignment	Differences in taxonomic classification tools and in the availability and quality of contig-level taxonomic information.	May result in incomplete or incorrect ARG–host associations, particularly for genes located on mobile genetic elements.
Validation strategy	Differences in the use of genomic, transcriptomic, proteomic, or phenotypic approaches to validate predicted resistance determinants.	Determines the extent to which predicted ARGs can be linked to gene expression, functional activity, or a resistant phenotype.

Note. The factors summarized above can act independently or interactively to influence ARG detection, abundance estimates, taxonomic assignment, and the comparability of resistome profiles across studies.

## Data Availability

No new data were created or analyzed in this study. Data sharing is not applicable to this article.
